# A systematic review of patient and clinician experiences of bariatric tourism

**DOI:** 10.1308/rcsann.2025.0020

**Published:** 2025-04-08

**Authors:** C Carey, S James, S Jaunoo

**Affiliations:** Conquest Hospital, East Sussex Healthcare NHS Trust, UK

**Keywords:** Health tourism, Bariatric surgery, Patient safety, General surgery

## Abstract

**Background:**

Although bariatric surgery is highly cost effective, accessing this treatment in the UK and other western nations is often challenging. Patients are therefore increasingly engaging in bariatric health tourism despite its associated risks and warnings from health institutions. A systematic review was performed to assess the reasons why patients are travelling abroad for surgery and common practices among bariatric tourism service providers.

**Methods:**

Medline and PubMed were searched for articles analysing the experiences of patients and service providers. Articles published in English between 2010 and 2023 were considered and seven were included for review after title, abstract and full text analysis.

**Results:**

Four studies assessing patients’ experiences and outcomes following bariatric tourism and three examining the perspectives of bariatric service providers were reviewed. Patients across the studies were mostly from western Europe, North America and the Middle East. The most common reasons for pursuing bariatric tourism were a lack of bariatric service provision in patients’ home nations, high costs of surgery in the private sector and long waiting times. Examples of practice outside the scope of most internationally recognised guidelines were identified across multiple studies, especially regarding patients’ pre-operative optimisation and follow-up. The studies therefore suggest that bariatric tourism may lead to significant health risks and issues that need managing once patients return home.

**Conclusions:**

Bariatric tourism is a common practice with significant associated risks. The reasons underpinning its appeal however, reflect genuine problems with accessing bariatric services and a lack of pre-operative education and optimisation.

## Introduction

Bariatric surgery is a complex but highly cost-effective method of managing obesity that is routinely offered to patients with a body mass index (BMI) greater or equal to 40kg/m^2^ or between 35 and 40kg/m^2^ alongside one or more serious conditions related to obesity.^1^ Patients should also be managed by a multidisciplinary team that can offer sufficient pre-operative optimisation and post-operative follow-up.^1^ Accessing bariatric surgery in the UK and other western countries, however, is often challenging due to long waiting times, perceptions that other treatment methods are more appropriate and strict patient selection criteria.^1,2^

Rising global rates of obesity^3^ alongside the challenges of providing bariatric surgery at scale have led many to opt for bariatric health tourism, during which patients travel abroad to undergo surgery.^2^ The availability of information online and aggressive marketing from bariatric tourism providers have also made accessing services abroad much easier.^4,5^ It is therefore likely that many will continue to consider bariatric tourism despite concerns raised by the British Obesity and Metabolic Surgery Society (BOMSS).^6^

Bariatric surgery is associated with significant rates of early and late complications even when all internationally recognised guidelines are followed.^7^ The cost of treating complications following bariatric tourism once patients return home may also be greater than the price of providing treatment locally.^2,8^ Making bariatric services more available may therefore convey significant clinical and financial benefits. The risks and rising prevalence of bariatric tourism highlight its growing importance and the need to understand its motivating factors. This systematic review analyses the reasons why patients pursue bariatric tourism and common practices among bariatric tourism providers.

## Methods

This review focused on studies that assessed the experiences and outcomes of bariatric tourists who travelled, or considered travelling, from more economically developed countries to less economically developed countries. Studies published in English between 2010 and 2023 that directly spoke to or surveyed bariatric patients and/or bariatric service providers were included. Inclusion also required the studies to describe the underlying reasons why patients engaged in bariatric tourism and/or outcomes following bariatric tourism, such as satisfaction rates, complications and follow-up practices. Case series, case reports and articles based on financial cost – benefit analyses and secondary data were excluded.

**Figure 1:**
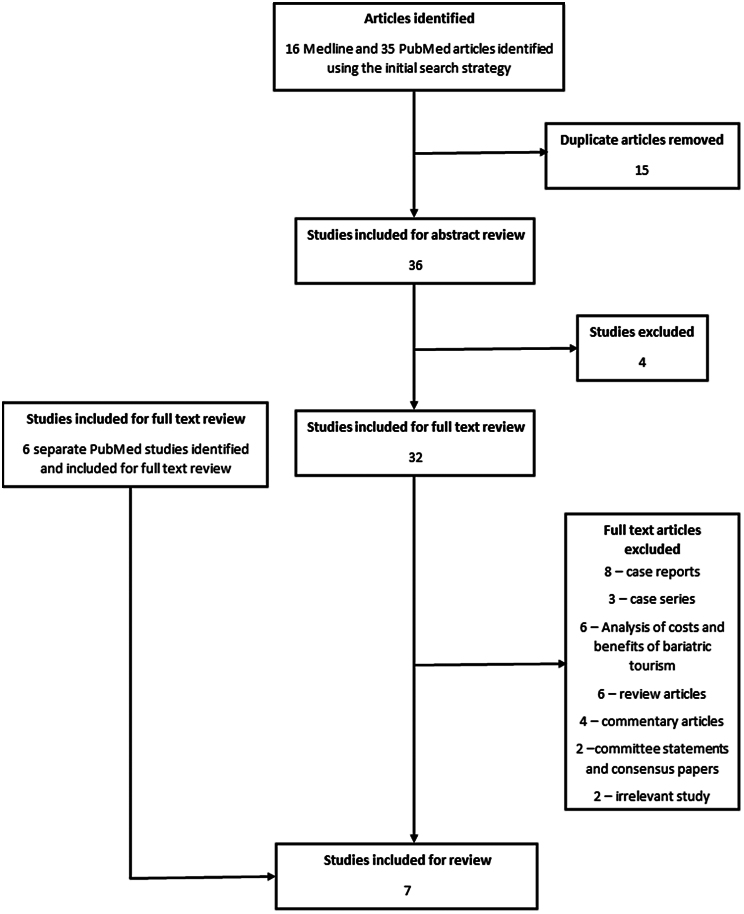
A flow chart that demonstrates how the studies were extracted using the search strategies and how they were systematically included and excluded after title, abstract and full text review.

The review was submitted and registered on PROSPERO (registration number CRD42025649021). Articles were found on Medline using the search strategies outlined in appendix Figures 1 and 2. The studies yielded were reviewed to determine whether they met the inclusion criteria. A total of 36 studies were initially found and 32 were included for full text review (Figure 1). A broader search for articles published after 2010 on PubMed was then performed using the MeSH terms ‘Bariatric surgery’ AND ‘Tourism’, and six additional studies were included for full text review (Figure 1). Seven studies that assessed the experiences of bariatric tourists and/or the practices of bariatric service providers were ultimately included for data extraction (Figure 1).

## Results

Four of the included articles focused on the views and outcomes described by bariatric patients and three assessed the practices of bariatric tourism providers. Table 1 displays the designs of the studies reviewed and the demographics of the patients assessed. Table 2 shows the criteria for recommending bariatric surgery in the patients’ countries of origin. The reasons for travelling abroad, procedures performed and follow-up practices outlined in the included studies are summarised in Table 3.

**Table 1:** Study designs and patient demographics

Authors (number of participants included)	Study design	Patients’ age	BMI	Patients’ country of origin	Country patients travelled to receive bariatric surgery
Jackson *et al*, 2018 (20)	Retrospective cross-sectional study	30–40: *n*=4 40–50: *n*=8 50–60: *n*=6	65% of participants spoke about how their BMI was too low to be considered for bariatric surgery in Canada	Canada	Mexico
Jackson *et al*, 2019 (20)	Retrospective cross-sectional study	30–40: *n*=4 40–50: *n*=8 50–60: *n*=6	65% of participants spoke about how their BMI was too low to be considered for bariatric surgery in Canada	Canada	Mexico
Choi *et al*, 2018 (12)	Qualitative	Average=45.35	Participants’ mean BMI was 50.1kg/m^2^.	Republic of Ireland	Not reported
Bashir *et al*, 2023 (78)	Retrospective cross-sectional study	Not reported	Not reported	Qatar (28.2%), Afghanistan (19.2%), UK (16.7%), UAE (11.5%), Saudi Arabia (9.0%), US (6.4%), Canada (3.8%) and Australia (1.3%)	Pakistan
Parmar *et al*, 2021 (277)	International survey of clinicians	Not reported	33.4% of surgeons believed there should be no BMI upper limit for bariatric surgery; 51.2% of surgeons believed bariatric surgery should not be offered to patients with a BMI >60kg/m^2^. 69.9% of surgeons believed that bariatric surgery should not be offered to patients with a BMI below 30–35kg/m^2^	32.0% of patients came from: US, Germany, UK, Saudi Arabia and Canada. The remaining 68.0% come from other unstated countries	52.3% of responding clinicians came from Brazil, Mexico, UK, US, Turkey, India, Argentina, Italy, Colombia, Germany and Spain. The remaining 47.7% came from 54 separate countries across all continents
Kowalewski *et al*, 2019 (64)	Retrospective online survey of bariatric surgeons	Not reported	Not reported	Most common origin countries (travelled to the Americas): Mexico and the US. Most common origin countries (travelled to the Middle East and Asia): UAE, Saudi Arabia and the US Most common origin countries (travelled to Europe): UK, Germany, Iraq, US and Canada Surgeons from India did not report a dominant origin	42.1% of surgeons came from: India (17.2%), Mexico (15.6%) and Turkey (9.4%) The remaining 57.9% came from 20 countries in North America, South America, Europe, Asia and Africa
Kowalewski *et al*, 2020 (6)	Retrospective online survey of bariatric surgeons	Not reported	Most foreign patients were indicated for surgery because their BMI was >40kg/m^2^ or >35kg/m^2^ with obesity related comorbidities. One patient was treated based on a BMI between 30–35kg/m^2^ as they had severe type 2 diabetes	UK, Germany, US, Republic of Ireland and Sweden	Poland

BMI = body mass index; UAE = United Arab Emirates

Study designs and demographics of the patients assessed across the studies. Common countries of origin included: the US, Canada, UK and Republic of Ireland. Many other patients came from Middle Eastern countries such as Saudi Arabia, UAE and Qatar. Patients commonly travelled to Mexico, India, Pakistan, Poland and Turkey for bariatric surgery.

**Table 2:** Common bariatric surgery practice in patients’ countries of origin and bariatric tourism destinations

Authors (number of patients included)	Bariatric surgery criteria in adult patients’ countries of origin	Bariatric surgery criteria where patients underwent surgery
Jackson *et al*, 2018 (20)	Canada follows the same guidance for recommending and managing bariatric surgical patients as NICE.^9^ Common procedures performed^9^ – Adjustable gastric banding – Sleeve gastrectomy – Gastric bypass	Mexico follows the same guidance for recommending and managing bariatric surgical patients as NICE.^10^ Common procedures performed^11^ – Gastric bypass – Sleeve gastrectomy
Jackson *et al*, 2019 (20)	The recommended practice in Canada is as described above	The recommended practice in Mexico is as described above
Choi *et al*, 2018 (12)	Ireland follows the same guidance for recommending and managing bariatric surgical patients as NICE.^12^ Common procedures performed^12^ – Laparoscopic gastric bypass—46% of procedures performed – Sleeve gastrectomy—46% of procedures performed	Not applicable
Bashir *et al*, 2023 (78)	The recommended practice in Canada is as described above. The UK, Saudi Arabia, US, Canada and Australia follow the same guidance for recommending and managing bariatric surgical patients as NICE. The most common procedures performed in these countries, Qatar and the UAE are^1,13–17^: – Sleeve gastrectomy – Roux-en-Y gastric bypass – Australia has also been noted as performing adjustable gastric band frequently Qatar—Bariatric surgery is offered to patients who meet the NICE criteria as well as for^13^: – Patients with a BMI 30–34.9kg/m^2^ with poorly controlled type 2 diabetes despite optimising medical and conservative measures – Bariatric surgery may be considered for South Asian patients with a BMI >27.5kg/m^2^ and type 2 diabetes – Patients in Qatar should receive specialist follow-up for at least two years and should have a specific long-term plan linked with primary care The most common bariatric procedures performed in the UAE and Qatar^13,^^17^: – Laparoscopic Roux-en-Y gastric bypass – Laparoscopic sleeve gastrectomy There is very little published information available for Pakistan and Afghanistan	Indications for bariatric surgery^18^: – Patients with a BMI >35 – Patients with a BMI >30 and at least one obesity-related disease In 2022, Abbas *et al* stated that bariatric surgery is still in its early stages and that laparoscopic procedures are performed on a relatively small scale in Pakistan, especially in the public sector. Laparoscopic sleeve gastrectomy and Roux-en-Y gastric bypass were noted as common procedures.^19^
Parmar *et al*, 2021 (277)	The recommended practice in the UK, US, Canada and Saudi Arabia is as described above. Germany follows the NICE criteria for bariatric surgery and recommends that patients with a BMI equal to or greater than 50 should also be offered bariatric surgery as an initial treatment.^20^ The common procedures in Germany include^21^: – Laparoscopic sleeve gastrectomy – Laparoscopic Roux-Y gastric bypass	Mexico and Turkey follow the same guidance for recommending and managing bariatric surgical patients as NICE^10,22^ India follows the International Federation for the Surgery of Obesity and Metabolic Disorders—Asia Pacific Chapter guidelines.^23,^^24^ Common procedures performed^11,24,25^ – Sleeve gastrectomy and gastric bypass were common in all three of the main travel destinations. – One anastomosis gastric bypass has been commonly reported in India – Biliopancreatic diversion has been commonly reported in Turkey
Kowalewski *et al*, 2019 (64)	The recommended practice in Mexico, the US, UAE, Saudi Arabia, UK, Germany and Canada is as described above. Iraq follows the same guidance for recommending and managing bariatric surgical patients as NICE.^26^ The most common procedures performed in Iraq^27^: – Laparoscopic sleeve gastrectomy – Laparoscopic mini gastric bypass	Poland follows the same guidance for recommending and managing bariatric surgical patients as NICE.^28^ Patients with a BMI >30kg/m^2^ who have had no response to medical methods of obesity treatment are also indicated for bariatric surgery. Common bariatric procedures performed^29^: – Laparoscopic sleeve gastrectomy – Laparoscopic Roux-en-Y gastric bypass – One anastomosis gastric bypass – Laparoscopic adjustable gastric banding
Kowalewski *et al*, 2020 (6)	The recommended practice in the UK, US, Ireland and Germany is as described above. Sweden follows the same guidance for recommending and managing bariatric surgical patients as NICE^30^. Common procedures performed in Sweden include^31^ – Laparoscopic sleeve gastrectomy and laparoscopic Roux-en-Y gastric bypass (93% of all procedures)	The recommended practice in Poland is as described above.

BMI = body mass index; UAE = United Arab Emirates

The recommended indications and management strategies after bariatric surgery in the patients’ countries of origin and travel destinations. This table and the wider analysis of the studies demonstrate that bariatric tourists often undergo procedures and receive care that is not in keeping with international guidelines and those followed in the countries where patients travel for surgery.

**Table 3:** Factors driving the desire for bariatric tourism and reported clinical practice

Authors (number of patients included)	Reasons for travelling abroad	Procedures performed	Follow-up practice
Jackson *et al*, 2018 (20)	Limited availability of surgery at home. BMI cut offs in the public sector. Long waiting times	Vertical sleeve gastrectomy (80%). Gastric plication (10%). Roux-en-Y gastric bypass (5%). Insertion of an adjustable lap band (5%)	Not reported
Jackson *et al*, 2019 (20)	Not assessed	Vertical sleeve gastrectomy (80.0%). Gastric plication (10.0%). Roux-en-Y gastric bypass (5.0%). Insertion of an adjustable lap band (5.0%)	Examples of follow-up practice included: Referral to a local bariatric service via their General Practitioner. General Practitioners encouraging follow-up with the foreign operating team. A lack of input from allied health professionals such as dieticians and psychiatrists. Failure to provide information about in-person support groups for people recovering after bariatric surgery. No follow-up on returning to Canada as information online was considered sufficient
Choi *et al*, 2018 (12)	A lack of self-control. To motivate them to live healthily. To improve quality of life. To maintain and improve family life. As a last resort	Not reported	Not reported
Bashir *et al*, 2023 (78)	Costs of surgery in home nations. Long pre-operative waiting times. Lack of social support patients could receive. Tourism. Advertisement. Privacy	Roux-en-Y gastric bypass (60.2%). Sleeve gastrectomy (39.8%)	Not reported
Parmar *et al*, 2021 (277)	Lower healthcare costs. Access to high-quality care. Long waiting times. Lack of bariatric service provision. Family support	Sleeve gastrectomy—offered by 81.6% of surgeons. Roux-en-Y gastric bypass—offered by 63.2%. Anastomosis/minigastric bypass (OAGB-MGB)—offered by 39.1%	51.7% of those surveyed felt that in-person follow-up was necessary. Around two-thirds were satisfied with virtual consultations
Kowalewski *et al*, 2019 (64)	High costs of surgery in local private healthcare systems. Long waiting times. The reputation of the individual surgeon. A lack of insurance coverage. A lack of any bariatric services	Laparoscopic sleeve gastrectomy—performed by 89.1% of respondents. Roux-en-Y gastric bypass—performed by 40.6% of respondents. Gastric bypass + anastomosis—performed by 37.5% of respondents	Follow-up longer than 3 months—77.8%. Follow-up 3 months post-operatively—4.8%. Follow-up 1 month post-operatively—4.8% Follow-up until stitches/clips were removed—12.7%
			Routine recommendation for follow-up by a local bariatric service—72.6%
			Face to face follow-up—54.7%. Video consultation follow-up—578%. Telephone consultation follow-up—70.3%. Email correspondence—78.1%. Discharge documents given in the patients’ native language—98.4%
Kowalewski *et al*, 2020 (6)	Not assessed	Laparoscopic sleeve gastrectomy—performed by 100%. Laparoscopic gastric bypass—performed by 100%. Mini gastric banding—performed by 66.7%. Laparoscopic gastric banding—performed by 33.3%. Single anastomosis duodenal ileal bypass with sleeve gastrectomy—performed by 16.7%. Duodenal switch—performed by 16.7%	Personal follow-up 3 months post-operatively—100%. Provision of discharge documents in English—83.3%. Recommendation of long-term follow-up in patients’ home country—83.3%

BMI = body mass index

The reasons why patients travelled abroad, the operations they underwent and common follow-up practices. The most common factors driving bariatric tourism were high costs, long waiting times and a lack of access to surgery in patients’ countries of origin. Common procedures performed included sleeve gastrectomy and Roux-en-Y gastric bypass. There was a lot of variation in the follow-up practices reported and many patients would have suffered complications that were managed and paid for within their countries of origin.

### Analysis of patient outcomes and opinions

Jackson *et al* performed a study assessing the experiences of 20 Canadian patients who travelled to Mexico for bariatric surgery.^32^ Of these, 80% underwent vertical sleeve gastrectomy, 10% underwent gastric plication and 5% underwent Roux-en-Y gastric bypass and laparoscopic adjustable gastric band insertion. The main reasons given for travelling abroad were: structural barriers to receiving surgery, the strict BMI cut offs needed to be considered suitable for surgery and long pre-operative treatment courses.^32^

All participants experienced structural difficulties when trying access bariatric surgery in Canada, with 60% citing lengthy periods between being accepted for surgery and their operation as a reason for travelling abroad.^32^ Many also wanted types of surgery that were difficult to access in Canada and others voiced concerns over the strict BMI criteria needed to be eligible for bariatric surgery and the quality of treatment available. Patients’ desire for agency over their care therefore appeared to drive methods of bypassing barriers to surgery.^32^

Jackson *et al* also performed a thematic analysis comparing the risks faced by Canadian bariatric tourists with those treated locally based on the same data set.^33^ Stigma and isolation from friends, family and clinicians were shown to drive patients to explore their options alone. These risks were compounded by difficulties associated with navigating the private healthcare system in Mexico without any help.^33^ A general lack of post-operative follow-up was also a significant risk, particularly when local clinicians showed reluctance to provide this care. A lack of pre-operative optimisation and informed consent for surgery were also highlighted.^33^

A qualitative study by Choi *et al* assessed the experiences of 12 Irish patients who sought bariatric services abroad.^34^ Many of these patients had not undergone surgery before the study. Patients often believed that surgery was an important motivator for their self-control and maintaining a healthier lifestyle. Long waiting times also negatively impacted patients’ physical and mental health and drove a desire for quicker treatment. The patients reported many barriers to bariatric tourism, including their personal finances, mobility issues and a lack of social support. There was also a lack of clear information about bariatric surgery abroad, which led to a lot of confusion and necessitated independent research.^34^

Formal bariatric tourism programs and greater financial support towards travelling to the UK for surgery were suggested as ways to improve bariatric tourism provision in Ireland.^34^ The respondents therefore appeared to favour travelling abroad to access care, but valued the need for regulation before and after their procedure. An integrated system in which patients could receive holistic bariatric care could theoretically help reduce the waiting lists for bariatric surgery significantly. Finding trusted centres for this in Europe, however, could prove challenging as many countries face the same issues with bariatric waiting lists as Ireland.

Bashir *et al* retrospectively assessed 78 patients who travelled to a single centre in Pakistan for bariatric surgery between 2018 and 2022.^35^ Thirteen patients travelled from the UK, five from the US and three from Canada; 92.3% reported high post-operative satisfaction and just 1.3% were dissatisfied following surgery. The most common reasons for travelling abroad were the cost of surgery locally (33.3%), long pre-operative waiting times (24.4%) and a lack of social support at home (21.8%). Tourism was cited by 6.4% and advertisement and privacy were cited by 5.1%. No patients travelled due to bariatric surgery being unavailable in their home nation or because the quality of surgery was greater in Pakistan.^35^

Although the authors concluded that Pakistan is a safe and viable destination for bariatric tourism, this is likely to have been influenced by selection and reporter bias as the study was conducted by clinicians working at the centre and not by independent researchers.^35^ Furthermore, patients who died or suffered major complications after surgery may have been less likely to be surveyed, which would have also led to selection bias.

### Studies assessing the opinions and experiences of clinicians

Parmar *et al* performed a survey of 277 clinicians from across the world about how they manage bariatric tourists.^36^ Most respondents were bariatric surgeons (92%) and others included dieticians, nurse specialists and other allied health care professionals. Patients’ most common countries of origin were: the US, Germany, the UK, Saudi Arabia and Canada. The cost of bariatric surgery led 77.3% of patients to pursue bariatric tourism and many felt that patients would travel to save 25–50% of their overall expense. Other less common factors included: a desire to access to high-quality care (40.3%), long waiting times (39.2%), a lack of local bariatric service provision (28.3%) and family support (14.4%).^36^

Significant minorities of clinicians felt that bariatric tourism carried major risks, including a higher chance of poor results (34.9%), complications (28.7%), a need for additional surgery (17.6%) and mortality (11.6%).^36^ Of the clinicians surveyed, 44.7% also felt that their patients had an inadequate level of pre-operative education and 18.3% reported that some had no work-up whatsoever; 51.7% believed that in-person follow-up was necessary and around two-thirds were satisfied with virtual consultations^36^; 13.6% even stated that the patient should be responsible for their own post-operative complications despite 85.1% acknowledging that bariatric tourism carried a risk of inadequate care after surgery. Parmar *et al* also suggest that patients may be unaware of important risks, as consent taking and the provision of a translator were not deemed necessary by 30.4% of respondents.^36^

Many respondents disagreed with the internationally recommended BMI cut offs for bariatric surgery and 33.4% believed that there should be no BMI cut off^1,36^; 51.2% stated that bariatric surgery should not be offered to patients with a BMI over 60kg/m^2^ and 69.9% stated that the lower BMI cut-off should be between 30 and 35kg/m^2^.^36^ Significant discrepancies were therefore shown between the quality of care bariatric patients can expect at home versus abroad. A similar study by Kowalewski *et al* examined the experiences of 64 surgeons from across the world with experience treating bariatric tourists.^37^ Most respondents came from India, Mexico and Turkey and the commonest procedures performed were laparoscopic sleeve gastrectomy, Roux-en-Y gastric bypass and gastric bypass with anastomosis formation.

Reasons cited for travelling abroad included high prices in private healthcare systems (54.7%), long waiting times in public healthcare systems (42.2%) and the reputation of individual surgeons (35.9%). A lack of either insurance coverage (37.5%) or bariatric services (31.2%) were also important motivators. Although a significant link was found between the cost of surgery and a country’s GDP per capita, the small size of this association may mean that the combined price of surgery and overseas travel may equal the cost of treatment locally.^37^ Instances of patients travelling from lower to higher income nations were also found, suggesting that cost was not the only motivating factor.^37^

Of the surgeons surveyed, 72.6% recommended that routine follow-up should take place in patients’ home countries.^37^ Although 77.8% followed patients up for more than three months, eight (12.7%) discharged patients after the surgical sutures/clips were removed. Fourteen (22.2%) reported post-operative complications, with five suffering venous thromboembolism and anastomotic leak and three experiencing ileus and internal bleeding.^37^ Since this study involved surgeons reporting their own complications and many followed up patients for short periods of time, it is highly likely that the rates of complications were higher than stated. The failure of operating teams to provide follow-up and high complication rate also highlight that bariatric tourism may pose major risks and expenses for local healthcare systems once patients return home.^37^

Another online survey by Kowalewski *et al* assessed the practice of six surgeons who managed 64 patients who travelled to Poland for bariatric surgery.^38^ Laparoscopic sleeve gastrectomy and laparoscopic bypass were performed by each of the surgeons and mini-gastric banding, gastric banding and duodenal switch were performed by four (66.7%), two (33.3%) and one (16.7%) surgeon respectively. The UK was again a common country of origin alongside Germany, the US, Ireland and Sweden. All surgeons carried out a three-month follow-up appointment and five (83.3%) recommended long-term follow-up in patients’ country of origin.^38^ This study therefore provides further evidence that a large amount of follow-up care following bariatric tourism is neglected by operating teams and so must take place in patients’ countries of origin.^38^

## Discussion

Two percent of bariatric operations worldwide are performed for bariatric tourists and, between 2020 and 2021, there were more British patients who underwent bariatric surgery abroad than with the NHS in England.^4,37^ A lack of bariatric service provision has also been noted in other western countries^32–34^ despite rises in the prevalence of obesity and type 2 diabetes.^39^ Bariatric tourism is therefore common, and healthcare systems must ensure that these patients are managed safely. Given the known health risks associated with obesity and the apparent lack of patient education regarding the risks of bariatric surgery,^6,7^ it is understandable why many in wealthier nations seek weight loss surgery abroad. Heavy marketing and package deals that help with transport and costs also allow patients to access services abroad with relative ease.^4,5^

The studies examined the real experiences reported by patients and clinicians who participate in bariatric tourism and this review builds our understanding of why patients travel abroad for surgery despite its risks. Long waiting times for surgery were described in four studies and drove patients to take control over their care and reduce their time living with obesity.^32,35–37^ Three studies also showed that the high cost of bariatric surgery in local private healthcare sectors was a strong motivational factor.^35–37^ Although allocating more funding to reduce waiting times for bariatric surgery may be challenging, the resources required to provide routine follow-up and manage complications after bariatric tourism can be extremely high. This study therefore suggests that British health providers should encourage patients to be treated locally to make long-term cost savings and ensure patient safety.^3,8^ Some patients also travelled abroad to improve their overall quality of life and because they could not access adequate social support at home.^34–36^ Improving these aspects of bariatric care in the UK may therefore also dissuade bariatric tourism and allow patients to return to work and other productive ventures more easily.

Instances of concerning practice were highlighted in many of the studies reviewed. A consistent lack of pre-operative optimisation from bariatric teams was striking given its importance to surgical outcomes.^33,36–38^ One example is that many of the bariatric tourists were unlikely to have switched to a low-energy diet before surgery, which has been shown to improve the view of the gastro-oesophageal region and significantly reduce the risk of surgical errors.^40^ A lack of face-to-face post-operative follow-up from operating teams was also widely reported.^33,36–38^ This increases the risk of poorly managing chronic complications after bariatric surgery, such as nutrient malabsorption, anastomotic strictures and fistula formation.^41^ These findings should allow British patients with little experience of foreign healthcare services to be better informed when choosing to travel abroad for surgery and help clinicians to provide the best possible care upon their return.

Greater standardisation and integration between bariatric tourism providers and local healthcare systems could help ensure that patients receive adequate care, as was suggested by Choi *et al.*^34^ This would require a major administrative effort and funding, however, that would probably equal or eclipse what is required to increase bariatric service provision locally. Others have suggested creating global registries of bariatric service providers to ensure that accreditation standards are maintained and that institutions are held to account when internationally recognised care standards are consistently not met.^35,42,43^ Once established, these registries could also help reduce the risks of bariatric tourism discussed in this review.

This study is limited by the small number of published studies reflecting the experiences of patients and clinicians with bariatric tourism. Additional studies from countries across the world would better illustrate the lived experiences of those who receive and perform bariatric surgery for foreign patients. In addition, the studies reviewed rely upon the self-reported experiences from participants, which may result in selection and response bias. This review did not formally compare the common outcomes seen by bariatric tourists versus those treated locally in the UK and other similar nations. Performing such a study would involve analysing national statistics and comparing quantitative and qualitative outcomes between bariatric tourists and patients treated locally. A comparison like this would be useful for patients considering bariatric tourism as it would allow its risks to be quantified and therefore better understood. Furthermore, it could help guide campaigns aimed at informing the public about the risks associated with bariatric tourism.

A financial analysis comparing the overall costs of bariatric surgery performed in the UK’s public and private sectors was also outside the scope of this study. An examination of the costs of bariatric surgery, its routine post-operative follow-up and of managing the early and late complications after bariatric tourism would allow governments to assess whether improving local bariatric services would be cost effective.

## Conclusions

This systematic review focused on the real-world experiences of bariatric tourists and bariatric service providers. Most patients travelled from higher to lower income countries and the commonest reasons for pursuing bariatric surgery abroad were long waiting times and high healthcare costs in patients’ home nations. Instances where international guidelines were not followed were common, particularly with respect to pre-operative optimisation and post-operative follow-up. Although this study outlines the common reasons why patients travel abroad for surgery, patients and clinicians should also acknowledge the frequent examples of concerning practice highlighted following bariatric tourism. The results should allow clinicians to counsel patients before they opt for bariatric tourism and encourage decision makers to develop more effective strategies of managing obesity in the UK.
